# Short-term effects of ambient PM_2.5_ on respiratory healthcare utilisation in older adults: a time-series study in an industrialising area of Central Thailand

**DOI:** 10.7189/jogh.16.04269

**Published:** 2026-09-04

**Authors:** Pirawan Wangupadcha, Yuparat Limmongkon, Steven Ronsmans, Lawrence Achilles Nnyanzi, Chananya Jirapornkul

**Affiliations:** 1 Faculty of Public Health, Khon Kaen University, Mueang Khon Kaen, Khon Kaen, Thailand; 2 Center for Environment and Health, Department of Public Health and Primary Care, KU Leuven, Leuven, Belgium; 3 School of Health and Life Sciences, Teesside University, Middlesbrough, UK

**Keywords:** PM_2.5_, air pollution, respiratory disease, elderly, time-series analysis, industrial area

## Abstract

**Background:**

Fine particulate matter (PM_2.5_) air pollution is a major environmental health concern and has been associated with adverse respiratory outcomes, particularly among older adults. Evidence regarding the short-term respiratory effects of PM_2.5_ in communities near newly industrialising areas in Thailand remains limited. We aimed to examine the short-term association between daily ambient PM_2.5_ concentrations and respiratory healthcare utilisation among older adults living near a newly established industrial area in central Thailand.

**Methods:**

We conducted a time-series study among residents aged ≥60 years living within a 5 km radius of an industrial area in central Thailand from November 2023 to March 2024. Daily respiratory healthcare visits (ICD-10 codes J00–J99) were obtained from electronic medical records of the local secondary-level hospital. We obtained daily PM_2.5_ concentrations and meteorological data from the nearest governmental air-quality monitoring station. Associations between PM_2.5_ exposure and respiratory visits were analysed using negative binomial regression models and expressed as incidence rate ratios (IRRs) per 10 μg/m^3^ increase in PM_2.5_. We used single-lag and moving-average models to assess delayed effects, adjusting for temperature, relative humidity, wind speed, day of the week, and temporal trends.

**Results:**

A total of 924 respiratory healthcare visits were recorded during the study period, with a mean of 6.37 visits per day. The mean daily PM_2.5_ concentration was 42.43 μg/m^3^ (standard deviation = 15.12), substantially exceeding the World Health Organization guideline. Positive associations between PM_2.5_ and respiratory visits were observed across several lag days, with the largest effect estimate at lag 4. A 10 μg/m^3^ increase in PM_2.5_ at lag 4 was associated with a 5.5% increase in respiratory healthcare visits (IRR = 1.055, 95% confidence interval = 0.995–1.119), although this association did not reach statistical significance. Subgroup analyses showed numerically larger effect estimates among females and individuals aged 60–69 years; however, interaction tests were not statistically significant.

**Conclusions:**

Short-term exposure to ambient PM_2.5_ showed a positive but non-significant association with respiratory healthcare utilisation among older adults living near a newly industrialising area. Continued efforts to improve air quality and protect vulnerable populations may help reduce pollution-related respiratory health burdens.

Air pollution remains one of the leading environmental risk factors for global morbidity and mortality [1,2]. Fine particulate matter (PM_2.5_) with an aerodynamic diameter ≤2.5 μm is of particular concern because its small size allows it to penetrate deep into the respiratory tract and translocate into the bloodstream, triggering systemic inflammation and oxidative stress [3–5].

Numerous epidemiological studies have demonstrated that short-term exposure to PM_2.5_ is associated with increased hospital visits, emergency department admissions, and mortality related to respiratory and cardiovascular diseases [6–8]. Older adults are especially susceptible to air pollution due to age-related declines in lung function, impaired immune responses, and a higher prevalence of chronic conditions [4,9].

Industrial area development has expanded significantly in central Thailand, as in many rapidly industrialising regions of Southeast Asia, leading to the establishment of communities near newly developed industrial estates to support economic growth. While industrialisation contributes to economic development, it may also increase environmental pollution and pose health risks to nearby populations residing in adjacent communities. However, evidence regarding the short-term health impacts of PM_2.5_ exposure in communities located near newly established industrial areas remains limited, particularly in Thailand.

Recent time-series studies from multiple countries have consistently reported short-term associations between PM_2.5_ exposure and respiratory health outcomes. However, despite the growing body of global evidence, studies examining the impacts within specific industrial micro-environments in developing countries remain very limited. For example, a time-series analysis conducted in Shijiazhuang, China reported a 0.72% increase in respiratory mortality per 10 μg/m^3^ increase in PM_2.5_ using a two-day moving average exposure window (lag 1) [10]. Similarly, pooled evidence from a large multi-city study covering 347 cities reported a 0.68% relative risk increase in respiratory mortality per 10 μg/m^3^ increase in PM_2.5_ using a two-day moving average model [7]. Studies in other regions have also demonstrated increased respiratory hospitalisations associated with short-term PM_2.5_ exposure, including a nationwide Brazilian study reporting a 3.28% increase in respiratory hospital admissions per 10 μg/m^3^ increase in PM_2.5_ using a distributed lag framework [11]. These findings suggest that short-term exposure to PM_2.5_ contributes to acute respiratory health effects across diverse geographic settings [10–12].

Understanding the acute health effects of PM_2.5_ in these settings is important for environmental health surveillance and public health interventions. Time-series analysis is commonly used to evaluate short-term associations between air pollution and health outcomes. Therefore, we aimed to examine the short-term association between daily ambient PM_2.5_ concentrations and respiratory healthcare utilisation among older adults living near a newly established industrial estate in central Thailand. We also evaluated lagged exposure effects and explored differences across demographic and health-related subgroups. We focused on communities within a 5 km radius of a newly established industrial estate, representing an early stage of industrial development where exposure patterns and population vulnerability may differ from those in long-standing industrial or urban settings. In Thailand, ambient PM_2.5_ concentrations are typically elevated during the November–March period because of regional haze, biomass burning, and mixed urban-industrial emissions. Accordingly, we evaluated the association between ambient PM_2.5_ exposure and respiratory healthcare utilisation in a vulnerable community setting rather than source-specific pollution effects.

## METHODS

### Study area and participants

We conducted a study in communities located within a 5 km radius of a newly established industrial area in central Thailand. The study area included communities under the service coverage of a district hospital responsible for the entire study area. The study population comprised older adults aged ≥60 years residing in the study area during the period from November 2023 to March 2024. This period corresponds to Thailand’s annual high-PM_2.5_ season, during which ambient particulate pollution is commonly elevated due to regional haze episodes, agricultural biomass burning, atmospheric inversion, and reduced atmospheric dispersion. We obtained data on respiratory healthcare utilisation from electronic medical records of the district hospital. Respiratory healthcare visits were identified using the ICD-10 codes J00–J99 and categorised into major respiratory disease groups, including acute upper respiratory infections (J00–J06), influenza and pneumonia (J09–J18), acute lower respiratory infections (J20–J22), other upper respiratory diseases (J30–J39), chronic lower respiratory diseases (J40–J47), and other respiratory diseases (J60–J99). We obtained environmental exposure data, including daily PM_2.5_ concentrations, from the nearest governmental air quality monitoring station, using it as a proxy for population-level exposure in the study area. We collected meteorological data, including temperature, relative humidity, and wind speed, for the same period.

### Data analysis

We conducted a retrospective time-series analysis to examine the short-term association between daily ambient PM_2.5_ concentrations and respiratory healthcare visits among older adults. The outcome variable was the daily count of respiratory healthcare visits.

We used negative binomial regression models with a log link function to account for overdispersion in count data. Effect estimates were expressed as incidence rate ratios (IRRs) with 95% confidence intervals (CIs) per 10 μg/m^3^ increase in PM_2.5_ concentration.

To evaluate short-term delayed and cumulative effects of PM_2.5_ exposure, we applied single-lag models (lag 0–5) and moving-average models (MA 2–6). Lag windows were pre-specified based on prior evidence of acute respiratory effects typically occurring within 0–5 days following exposure. These exposure windows were selected to capture both immediate and short-term delayed associations without imposing additional functional assumptions on the exposure-lag relationship.

We adjusted all models for potential time-varying confounders, including daily temperature, relative humidity, wind speed, and day of the week. Long-term and seasonal trends during the study period were controlled using a natural cubic spline function of time, with degrees of freedom selected based on model fit assessed using the Akaike information criterion and inspection of residual diagnostics.

We conducted subgroup analyses to assess potential effect modification by gender, age group, presence of chronic disease, and body mass index. Interaction terms were included in the regression models to formally test statistical differences between subgroups.

Given that multiple lag structures were evaluated, lag-specific results were considered exploratory. We did not apply formal adjustment for multiple comparisons due to the high correlation between lag estimates; however, we acknowledged the potential for type I error inflation in interpretation. An offset term for population size was not included due to the relatively stable population within the defined catchment area over the study period.

We used Stata, version 15.0 (StataCorp LLC, College Station, Texas, USA) for all analyses. Statistical significance was defined as a two-sided *P*-value <0.05

## RESULTS

### Characteristics of respiratory healthcare visits

During the study period (November 2023 to March 2024), a total of 924 respiratory healthcare visits were recorded among older adults aged ≥60 years living in communities near the industrial area, with an average of 6.37 visits per day (**Table 1**). Most visits occurred among individuals aged 60–69 years, followed by those aged 70–79 years and ≥80 years. Visits were equally distributed between females and males. Chronic lower respiratory diseases and acute upper respiratory infections accounted for the largest proportion of respiratory healthcare visits.

**Table 1:** Baseline characteristics of respiratory healthcare visits among older adults (n = 924)

	n (%)
**Respiratory tract diseases**	
Chronic lower respiratory diseases (J40–J47)	310 (33.55)
Acute upper respiratory infections (J00–J06)	281 (30.41)
Other upper respiratory diseases (J30–J39)	139 (15.04)
Influenza and pneumonia (J09–J18)	106 (11.47)
Acute lower respiratory infections (J20–J22)	46 (4.98)
Other respiratory diseases (J60–J99)	42 (4.55)
**Gender**	
Female	462 (50.00)
Male	462 (50.00)
**Age (years)**	
60–69	449 (48.92)
70–79	307 (32.90)
≥80	168 (18.18)
x̄ (SD)	71.13 (7.67)
Mdn (Min–Max)	70 (60–97)
**BMI (kg/m^2^)**	
Underweight (<18.50)	154 (16.67)
Normal weight (18.50–22.99)	356 (38.53)
Overweight (≥23.00)	414 (44.80)
x̄ (SD)	22.28 (4.58)
Mdn (Min–Max)	22.27 (12.94–45.01)
**Chronic disease (DM and/or hypertension)**	
No	681 (73.70)
Yes	243 (26.30)

BMI – body mass index, DM – diabetes mellitus, Max – maximum, Mdn – median, Min – minimum, SD – standard deviation, x̄ – mean

### Environmental conditions during the study period

Daily PM_2.5_ concentrations and meteorological conditions varied during the study period (**Table 2**). The mean daily PM_2.5_ concentration was 42.43 μg/m^3^ (standard deviation = 15.12), substantially exceeding the World Health Organization 24-hour air quality guideline of 15 μg/m^3^. Daily PM_2.5_ levels showed considerable temporal variation during the study period. Meteorological conditions were relatively stable overall. The mean relative humidity during the study period was 72.06% (standard deviation = 14.29), while daily temperature and wind speed exhibited typical seasonal fluctuations for the region. Relative humidity was included in the regression models as a potential meteorological confounder.

**Table 2:** Descriptive statistics of daily environmental variables during the study period

	x̄ (SD)	Min	Max
**PM_2.5_ (μg/m^3^)**	42.43 (15.12)	22.05	96.81
**Temperature (°C)**	28.97 (1.09)	24.20	31.34
**Wind speed (m/s)**	1.74 (0.58)	0.71	3.15
**Relative humidity (%)**	72.06 (14.29)	31.29	95.87
**Daily respiratory visits**	6.37 (4.08)	1	25

Max – maximum, Min – minimum, PM_2.5_ – fine particulate matter, SD – standard deviation, x̄ – mean

### Association between PM_2.5_ and respiratory healthcare visits

In the single-lag models, minor variations in effect estimates were observed across lag days (**Table 3**). The largest estimate was found at lag 4, where a 10 μg/m^3^ increase in PM_2.5_ was associated with a 5.5% increase in respiratory healthcare visits (IRR = 1.055, 95% CI = 0.995–1.119). However, all lag-specific CIs included the null value, indicating that none of the associations were statistically significant.

**Table 3:** Association between PM_2.5_ exposure and respiratory outpatient visits (single-lag models)

Exposure lag (PM_2.5_ per 10 μg/m^3^)	IRR (95% CI)	*P*-value
Lag 0	1.020 (0.980–1.060)	0.550
Lag 1	1.012 (0.970–1.050)	0.350
Lag 2	1.011 (0.960–1.060)	0.350
Lag 3	1.027 (0.990–1.070)	0.084
Lag 4	1.055 (0.995–1.119)	0.073
Lag 5	1.024 (0.960–1.090)	0.430

CI – confidence interval, IRR – incidence rate ratio, PM_2.5_ – fine particulate matter

Moving-average models (MA 2–6) were applied to assess potential cumulative short-term effects of PM_2.5_ exposure. The effect estimates were close to unity (IRRs = 0.999–1.002), and all CIs included the null value, indicating no statistically significant cumulative association between PM_2.5_ exposure and respiratory healthcare utilisation over 2–6-day exposure windows (Table S1 in the **Online Supplementary Document**).

Overall, findings from both single-lag and moving-average models indicate no statistically significant association between short-term PM_2.5_ exposure and respiratory healthcare visits in this study population. Although the highest point estimate was observed at lag 4, this should be interpreted cautiously, as it may reflect random variation arising from multiple lag specifications rather than a true exposure-response relationship.

Given the exploratory nature of lag selection and the evaluation of multiple exposure windows, variability in effect estimates across lag days is expected. No formal adjustment for multiple comparisons was applied; therefore, chance findings across lag structures cannot be excluded.

**Figure 1:**
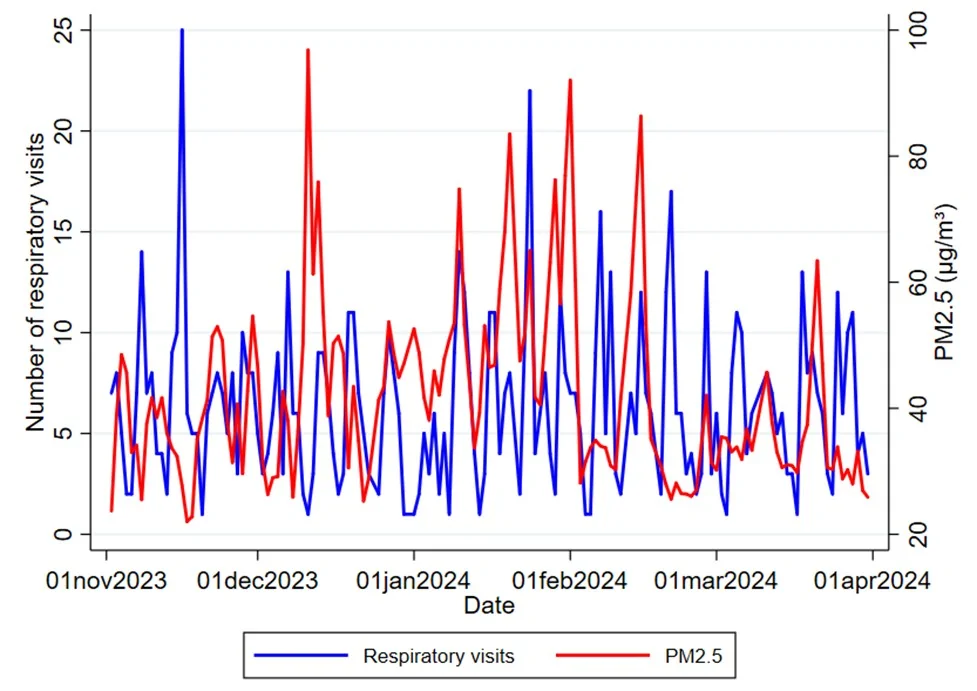
Time-series of daily PM_2.5_ concentrations and respiratory outpatient visits in the study area from November 2023 to March 2024. PM_2.5_ – fine particulate matter.

Temporal trends of daily PM_2.5_ concentrations and respiratory healthcare visits during the study period were observed (**Figure 1**). Daily PM_2.5 _concentrations and respiratory healthcare visits showed substantial day-to-day variability throughout the study period. PM_2.5 _concentrations fluctuated between approximately 22 and 97 μg/m³, with several pronounced peaks, particularly during December 2023 and January–February 2024. Daily respiratory healthcare visits also varied considerably, ranging from 1 to 25 visits per day, with intermittent peaks occurring throughout the study period. Although increases in respiratory visits occasionally occurred during or near periods of elevated PM_2.5_ concentrations, the temporal patterns of the two series did not consistently coincide, suggesting that any short-term association could not be determined from visual inspection alone.

**Figure 2:**
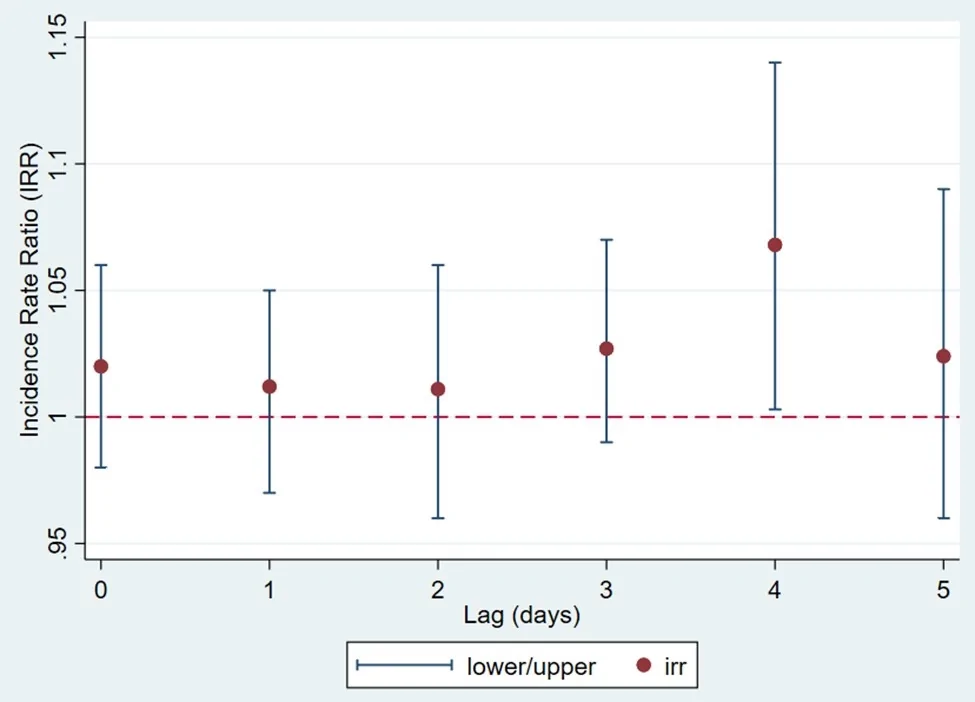
Lag-response association between PM_2.5_ exposure and daily respiratory outpatient visits. PM_2.5_ – fine particulate matter.

The lag-response pattern showed modest variation in the estimated association between PM2.5 exposure and respiratory healthcare visits across lag 0–5 days (**Figure 2**). The IRRs were above 1.00 at all examined lag days, with estimates increasing from lag 2 to lag 4 and reaching the highest value at lag 4 (IRR = 1.055; 95% CI = 0.995–1.119), before decreasing at lag 5. However, the 95% CIs included the null value of 1.00 at all lag days, indicating that none of the lag-specific associations reached statistical significance.

The association appeared slightly stronger among females compared with males. Similarly, individuals aged 60–69 years showed somewhat higher effect estimates than older age groups. However, formal interaction tests between PM_2.5_ exposure and subgroup variables were not statistically significant, indicating that these differences should be interpreted cautiously (Table S2 in the **Online Supplementary Document**).

## DISCUSSION

In this time-series study, we observed a positive but non-significant association between ambient PM_2.5_ exposure and respiratory healthcare utilisation among older adults living near a newly established industrial area in central Thailand. The largest effect estimate was observed at lag 4, suggesting a possible delayed respiratory response following PM_2.5_ exposure.

Our findings are consistent with a growing body of epidemiological evidence demonstrating that short-term exposure to PM_2.5_ increases respiratory morbidity and healthcare utilisation [11,13–15]. Large multi-city studies have reported similar associations between daily PM_2.5_ concentrations and hospital admissions for respiratory diseases across diverse geographic regions [7]. For example, a nationwide time-series analysis in China reported that short-term increases in PM_2.5_ were significantly associated with higher hospital admission rates among adults, particularly among elderly populations [6,16].

Several individual time-series studies have also reported comparable effect estimates. A time-series analysis conducted in Shijiazhuang, China reported a 0.72% increase in respiratory mortality per 10 μg/m^3^ increase in PM_2.5_ using a two-day moving average exposure window [10]. Similarly, pooled evidence from a large multi-city study covering 347 cities reported a 0.68% increase in respiratory mortality associated with a 10 μg/m^3^ increase in PM_2.5_ [7]. In Brazil, a nationwide study focusing on elderly populations reported a 3.28% increase in respiratory hospital admissions per 10 μg/m^3^ increase in PM_2.5_ using a distributed lag modelling approach [11]. In addition, studies conducted in Lanzhou and Changsha, China, have reported positive associations between PM_2.5_ exposure and respiratory morbidity, with relative risks around 1.019–1.030 per 10 μg/m^3^ increase in PM_2.5_ depending on the lag structure examined [12,15]. Although direct comparisons across studies should be made cautiously because outcome definitions differed (*e.g.* healthcare utilisation *vs* mortality), the direction of association was consistent with previous evidence linking short-term PM_2.5_ exposure to adverse respiratory outcomes. Several factors may explain the larger effect size we observed. First, the study population consisted exclusively of older adults, who are more susceptible to air pollution because of age-related physiological decline and a higher prevalence of chronic conditions. Second, the study area is located near a newly established industrial area, where localised emissions and potentially higher pollutant toxicity may contribute to stronger health effects than in more heterogeneous urban settings. However, we did not design this study to identify specific PM_2.5_ sources, and ambient pollution likely reflected combined contributions from industrial emissions, traffic, regional haze, biomass burning, and seasonal atmospheric conditions. Therefore, the findings should be interpreted as health effects of ambient PM_2.5_ in a mixed-source environment rather than industrial emissions alone. Third, methodological differences in exposure assessment, lag structures, and outcome definitions may influence effect estimates. Additionally, relatively high baseline PM_2.5_ concentrations in the study area may have amplified short-term health impacts.

The delayed effect we observed is biologically plausible. Due to its small size, PM_2.5_ can penetrate deep into the respiratory tract and induce airway inflammation, oxidative stress, and impaired pulmonary immune responses [2,4,5,17,18]. These pathophysiological processes may take several days to develop before leading to clinically significant respiratory symptoms requiring healthcare utilisation, particularly among individuals with chronic lower respiratory diseases or acute respiratory infections. Previous studies have similarly reported lag structures ranging from 0 to 4 days between PM exposure and respiratory hospitalisations [13–15]. In the present study, chronic lower respiratory diseases (J40–J47) and acute upper respiratory infections (J00–J06) accounted for the largest proportion of respiratory healthcare visits, supporting the biological plausibility of the observed respiratory outcomes. In addition, a short delay between symptom onset and healthcare utilisation may also contribute to the observed lag structure, as individuals may not seek medical care immediately when symptoms first appear. However, because the outcome definition included heterogeneous respiratory diagnoses grouped under ICD-10 codes J00–J99, the overall effect estimate may have been attenuated by combining respiratory conditions with varying susceptibility to PM_2.5_ exposure.

Ambient PM_2.5_ concentrations observed during the study period were relatively high, with an average concentration of 42.43 μg/m^3^, substantially exceeding the World Health Organization recommended 24-hour guideline of 15 μg/m^3^ [19]. Elevated particulate pollution levels in the study area are consistent with previous epidemiological evidence from the same region. A previous study conducted in communities surrounding the same industrial area reported increased risks of hospitalisation for lower respiratory tract illness among elderly residents, suggesting that populations living near the industrial complex may experience elevated respiratory health risks associated with particulate pollution exposure [9,20]. Analysing temporal variation in daily PM_2.5_ concentrations and respiratory healthcare visits during the study period, we found fluctuations in PM_2.5_ levels over time, with several periods of elevated concentrations coinciding with increases in respiratory healthcare visits (**Figure 1**). Although the ecological nature of the analysis does not allow causal inference at the individual level, the temporal patterns provide supportive evidence for the short-term association identified in the statistical models.

Numerous daily time-series studies have examined both immediate and delayed effects of PM_2.5_ exposure using single-day lag models and cumulative lag structures. Several studies have reported the strongest associations within the first 1–2 days after exposure [10,12,20], suggesting rapid triggering effects of particulate pollution on respiratory events. However, other studies have identified delayed peaks at later lag days, such as lag 6 or lag 7 [15], indicating that inflammatory and physiological responses may continue to evolve for several days following exposure. In addition to single-day lags, cumulative exposure windows (*e.g.* moving averages of 2–4 days or cumulative lag 0–7 models) often yield larger or more robust effect estimates, suggesting short-term accumulation of risk [14,21]. In this study, we also examined moving-average exposure models (MA 2–6) to assess cumulative short-term effects of PM_2.5_ exposure. However, the associations observed in these cumulative models were small and did not reach statistical significance, suggesting that the short-term respiratory impacts in this population were more strongly associated with a specific delayed lag rather than cumulative exposure over several days. The delayed peak we observed at lag 4 is therefore consistent with previously reported lag patterns and may reflect the time required for airway inflammation and exacerbation of respiratory conditions following PM_2.5_ exposure [14,22]. We also found a lag-response relationship between PM_2.5_ exposure and respiratory healthcare visits (**Figure 2**), with estimated IRRs gradually increasing across lag days and reaching the highest point at lag 4 before declining at later lags. This pattern supports the interpretation that the respiratory health impacts of PM_2.5_ exposure may occur with a short delay rather than immediately following exposure.

Previous epidemiological studies have identified several potentially vulnerable subgroups with elevated respiratory risks following PM_2.5_ exposure. Older adults are consistently reported as one of the most susceptible populations due to age-related declines in pulmonary function and a higher prevalence of chronic diseases [11]. Some studies have also reported stronger associations among males or during cold seasons when air pollution levels are often higher and respiratory infections are more prevalent [15,21]. In this study, subgroup analyses of effect estimates were numerically higher among females and individuals aged 60–69 years, although interaction tests were not statistically significant. Variability in subgroup findings has been widely reported in previous studies and may reflect differences in population characteristics, exposure patterns, and local environmental conditions [11,23].

The study area is located near a rapidly developing industrial area, where communities may experience combined exposure from industrial emissions, traffic-related pollution, and regional haze episodes. Rapid industrialisation across many parts of Southeast Asia has raised concerns regarding environmental pollution and its potential health impacts. Populations living near industrial complexes may experience elevated exposure to multiple pollution sources, which could increase respiratory health risks. Our findings therefore provide empirical evidence supporting the need for enhanced environmental monitoring and public health surveillance in industrialising regions.

Although the overall evidence linking PM_2.5_ exposure with respiratory health outcomes is consistent, heterogeneity in effect estimates has been reported across studies. Differences in exposure assessment methods, baseline pollution levels, population susceptibility, and healthcare utilisation patterns may contribute to variation in observed effect sizes [14,22]. In addition, exposure misclassification resulting from spatial differences between monitoring stations and study populations may attenuate effect estimates in some settings [14,23]. Nevertheless, most epidemiological studies continue to report positive associations between PM_2.5_ exposure and respiratory morbidity and mortality. Although short-term exposure to low concentrations of ambient air pollution has been significantly associated with increased respiratory outpatient visits [9,18,20].

This study has several strengths. First, the time-series design enabled the evaluation of short-term associations while inherently controlling for individual characteristics that remain constant over time. Second, routinely collected hospital data provided real-world evidence of healthcare utilisation in a community setting. We also evaluated multiple lag structures to explore potential delayed respiratory responses following PM_2.5_ exposure. Because several lag models were examined, the statistically significant association observed at lag 4 may partly reflect multiplicity and should be interpreted cautiously. Nevertheless, similar delayed lag structures have been reported in previous air pollution studies, supporting the biological plausibility of delayed respiratory responses following PM_2.5_ exposure.

However, several limitations should be considered. First, we obtained PM_2.5_ exposure from a monitoring station located approximately 15–20 km from the study community, which may introduce exposure misclassification. Nevertheless, previous studies have demonstrated that PM_2.5_ exhibits relatively stable spatial patterns and can be reasonably estimated using area-level monitoring data [24,25]. Additionally, the ecological study design does not allow assessment of individual-level exposure, and information on potential confounders such as indoor air pollution and personal protective behaviours were unavailable. Respiratory healthcare utilisation may also be influenced by healthcare-seeking behaviour, healthcare accessibility, public holidays, and provider-side factors that could not be fully accounted for using secondary data. Because we used anonymised hospital records, repeated visits by the same individuals could not be identified. The relatively short study duration may limit the ability to capture long-term seasonal variability and may reduce statistical power. Furthermore, the respiratory outcome definition included a broad range of ICD-10 respiratory diagnoses (J00–J99), encompassing heterogeneous respiratory conditions with potentially different aetiologies and susceptibilities to PM_2.5_ exposure. Therefore, diagnostic heterogeneity should be considered when interpreting the findings, particularly because both acute and chronic respiratory conditions were included in the outcome definition.

Our findings highlight the importance of strengthening air quality management and environmental health surveillance in rapidly industrialising areas. Early warning systems, pollution control strategies, and targeted protection of vulnerable populations – particularly older adults – should be prioritised to reduce pollution-related respiratory health impacts.

## CONCLUSIONS

Short-term increases in ambient PM_2.5_ concentrations were suggestively associated with respiratory healthcare utilisation among older adults. We observed the highest estimate at lag 4; however, the association was not statistically significant and should be interpreted cautiously. These findings should be considered exploratory and require further confirmation.

## Additional material


Online Supplementary Document

## References

[1] Burnett R Chen H Szyszkowicz M Fann N Hubbell B Pope CA III Global estimates of mortality associated with long-term exposure to outdoor fine particulate matter. Proc Natl Acad Sci U S A. 2018;115:9592–7. 10.1073/pnas.1803222115 30181279 PMC6156628

[2] Cohen AJ Brauer M Burnett R Anderson HR Frostad J Estep K Estimates and 25-year trends of the global burden of disease attributable to ambient air pollution: an analysis of data from the Global Burden of Diseases Study 2015. Lancet. 2017;389:1907–18. 10.1016/S0140-6736(17)30505-6 28408086 PMC5439030

[3] Bhatnagar A Cardiovascular Effects of Particulate Air Pollution. Annu Rev Med. 2022;73:393–406. 10.1146/annurev-med-042220-011549 34644154 PMC10132287

[4] Rajagopalan S Al-Kindi SG Brook RD Air Pollution and Cardiovascular Disease: JACC State-of-the-Art Review. J Am Coll Cardiol. 2018;72:2054–70. 10.1016/j.jacc.2018.07.099 30336830

[5] Thangavel P Park D Lee YC Recent Insights into Particulate Matter (PM2.5)-Mediated Toxicity in Humans: An Overview. Int J Environ Res Public Health. 2022;19:7511. 10.3390/ijerph19127511 35742761 PMC9223652

[6] Liu H Tian Y Xiang X Juan J Song J Cao Y Ambient Particulate Matter Concentrations and Hospital Admissions in 26 of China’s Largest Cities: A Case-Crossover Study. Epidemiology. 2018;29:649–57. 10.1097/EDE.0000000000000869 29870428

[7] Orellano P Reynoso J Quaranta N Bardach A Ciapponi A Short-term exposure to particulate matter (PM(10) and PM(2.5)), nitrogen dioxide (NO(2)), and ozone (O(3)) and all-cause and cause-specific mortality: Systematic review and meta-analysis. Environ Int. 2020;142:105876. 10.1016/j.envint.2020.105876 32590284

[8] Wei Y Wang Y Di Q Choirat C Wang Y Koutrakis P Short term exposure to fine particulate matter and hospital admission risks and costs in the Medicare population: time stratified, case crossover study. BMJ. 2019;367:l6258. 10.1136/bmj.l6258 31776122 PMC6880251

[9] Wangupadcha P Ronsmans S Vanoirbeek J Limmongkon Y Phokee W Jirapornkul C Increased Risk of Hospitalization for Lower Respiratory Tract Illness (LRTI) in the Elderly Living in Communities near a Newly Established Industrial Estate in Central Thailand. Int J Environ Res Public Health. 2025;22:874. 10.3390/ijerph22060874 40566301 PMC12193676

[10] Guan M Sun C Tang DG Kang H Chen F A Time-Series Analysis on the Association Between Fine Particulate Matter and Daily Mortality — Shijiazhuang City, Hebei Province, China, 2015–2020. China CDC Wkly. 2022;4:226–31. 35433077 10.46234/ccdcw2022.052 PMC9005476

[11] Ferreira AP Ferreira PS Silva AMC Air pollution by fine particulate material on hospitalization for respiratory diseases in the elderly. Rev Contemp. 2024;4:e3867. 10.56083/RCV4N4-013

[12] Feng Q Chen Y Su S Zhang X Lin X Acute effect of fine particulate matter and respiratory mortality in Changsha, China: a time-series analysis. BMC Pulm Med. 2022;22:416. 10.1186/s12890-022-02216-3 36368963 PMC9652800

[13] Hu J Yu L Yang Z Qiu J Li J Shen P Long-Term Exposure to PM(2.5) and Mortality: A Cohort Study in China. Toxics. 2023;11:727. 10.3390/toxics11090727 37755738 PMC10534778

[14] Liu C Chen R Sera F Vicedo-Cabrera AM Guo Y Tong S Ambient Particulate Air Pollution and Daily Mortality in 652 Cities. N Engl J Med. 2019;381:705–15. 10.1056/NEJMoa1817364 31433918 PMC7891185

[15] Pothirat C Chaiwong W Liwsrisakun C Bumroongkit C Deesomchok A Theerakittikul T Acute effects of air pollutants on daily mortality and hospitalizations due to cardiovascular and respiratory diseases. J Thorac Dis. 2019;11:3070–83. 10.21037/jtd.2019.07.37 31463136 PMC6687987

[16] Tian Y Liu H Wu Y Si Y Song J Cao Y Association between ambient fine particulate pollution and hospital admissions for cause specific cardiovascular disease: time series study in 184 major Chinese cities. BMJ. 2019;367:l6572. 10.1136/bmj.l6572 31888884 PMC7190041

[17] Li G Huang J Xu G Pan X Qian X Xu J The short term burden of ambient fine particulate matter on chronic obstructive pulmonary disease in Ningbo, China. Environ Health. 2017;16:54. 10.1186/s12940-017-0253-1 28587653 PMC5461635

[18] Lei J Chen R Liu C Zhu Y Xue X Jiang Y Fine and coarse particulate air pollution and hospital admissions for a wide range of respiratory diseases: a nationwide case-crossover study. Int J Epidemiol. 2023;52:715–26. 10.1093/ije/dyad056 37159523

[19] World Health Organization. WHO global air quality guidelines: particulate matter (PM2.5 and PM10), ozone, nitrogen dioxide, sulfur dioxide and carbon monoxide. Geneva, Switzerland: World Health Organization; 2021. Available: https://www.who.int/publications/i/item/9789240034228. Accessed: 1 May 2026. 34662007

[20] Sacramento DS Martins LC Arbex MA Pamplona YAP Atmospheric Pollution and Hospitalization for Cardiovascular and Respiratory Diseases in the City of Manaus from 2008 to 2012. ScientificWorldJournal. 2020;2020:8458359. 10.1155/2020/8458359 32308570 PMC7152981

[21] Sun Y Milando CW Spangler KR Wei Y Schwartz J Dominici F Short term exposure to low level ambient fine particulate matter and natural cause, cardiovascular, and respiratory morbidity among US adults with health insurance: case time series study. BMJ. 2024;384:e076322. 10.1136/bmj-2023-076322 38383039 PMC10879982

[22] Huang JY Feng W Sang GX McDonald S He TF Lin Y Association between short-term exposure to ambient air pollutants and the risk of hospital visits for acute upper respiratory tract infections among adults: a time-series study in Ningbo, China. BMC Public Health. 2024;24:1555. 10.1186/s12889-024-19030-7 38858655 PMC11163729

[23] Jacobs JH Strak M Velders GJM Zorn J Hogerwerf L Simões M Short-term exposure to ambient air pollution and severe COVID-19: mortality and hospital admission to COVID-19 in the Netherlands from February to December 2020. Environ Adv. 2024;17:100592. 10.1016/j.envadv.2024.100592

[24] Di Q Amini H Shi L Kloog I Silvern R Kelly J An ensemble-based model of PM(2.5) concentration across the contiguous United States with high spatiotemporal resolution. Environ Int. 2019;130:104909. 10.1016/j.envint.2019.104909 31272018 PMC7063579

[25] Shen Y Yao L PM2.5, Population Exposure and Economic Effects in Urban Agglomerations of China Using Ground-Based Monitoring Data. Int J Environ Res Public Health. 2017;14:716. 10.3390/ijerph14070716 28671643 PMC5551154

