# Supplementary material for: Short-term effects of ambient PM2.5 on respiratory healthcare utilisation in older adults: a time-series study in an industrialising area of Central Thailand
Source: J Glob Health. 2026 Sep 4;16:04269. doi: 10.7189/jogh.16.04269 (PMC13542799; doi:10.7189/jogh.16.04269)
Supplement: Supplementary file 1 [file jogh-16-04269-s001.pdf]

Supplement to: Wangupadcha P, Limmongkon Y, Ronsmans S, Nnyanzi LA, Jiraponkul C. Short-term effects of ambient PM2.5 on respiratory healthcare utilisation in older adults: a time-series study in an industrialising area of Central Thailand. J Glob Health. 2026;16:04269.

Table S1. Association between moving-average PM2.5 exposure and respiratory outpatient visits per 10 µg/m³ increase

Moving average	IRR	95% CI	p-value
MA 2 (0–1)	0.999	0.991–1.007	0.884
MA 3 (0–2)	1.001	0.994–1.009	0.774
MA 4 (0–3)	1.002	0.994–1.009	0.661
MA 5 (0–4)	1.002	0.993–1.010	0.705
MA 6 (0–5)	1.002	0.993–1.010	0.705

CI – confidence interval, IRR – incidence rate ratio, $PM_{2.5}$ – particulate matter with an aerodynamic diameter ≤2.5 µm

Table S2. Subgroup analysis of the association between PM2.5 exposure (lag 4) and respiratory visits

Subgroup	IRR per 10 µg/m³	95% CI	p-value (main effect)	p-value (interaction)
Gender				0.359
Female	1.079	1.000–1.164	0.049	
Male	1.059	0.987–1.137	0.112	
Chronic disease				0.834
No chronic	1.059	0.991–1.132	0.088	
Chronic	1.045	0.965–1.132	0.321	
Age group				0.199
60–69 years	1.126	1.041–1.219	0.003	
≥70 years	1.092	0.998–1.193	0.054	
BMI category				0.760
<23 kg/m²	1.044	0.965–1.129	0.281	
≥23 kg/m²	1.061	0.984–1.143	0.121	

Estimates were obtained from negative binomial regression models with interaction terms between $PM_{2.5}$ (lag 4) and subgroup variables, adjusted for temperature, wind speed, and day of the week.

BMI – body mass index, CI – confidence interval, IRR – incidence rate ratio, $PM_{2.5}$ – particulate matter with an aerodynamic diameter ≤2.5 µm
